# Interferon-Based Therapy Decreases Risks of Hepatocellular Carcinoma and Complications of Cirrhosis in Chronic Hepatitis C Patients

**DOI:** 10.1371/journal.pone.0070458

**Published:** 2013-07-23

**Authors:** Ching-Sheng Hsu, Chun-Jen Huang, Jia-Horng Kao, Hans Hsienhong Lin, You-Chen Chao, Yen-Chun Fan, Pei-Shan Tsai

**Affiliations:** 1 Division of Gastroenterology, Department of Internal Medicine, Buddhist Tzu Chi General Hospital, Taipei Branch, Taipei, Taiwan; 2 School of Medicine, Tzu Chi University, Hualien, Taiwan; 3 Department of Anesthesiology, Buddhist Tzu Chi General Hospital, Taipei Branch, Taipei, Taiwan; 4 School of Medicine, Taipei Medical University, Taipei, Taiwan; 5 Graduate Institute of Clinical Medicine, National Taiwan University College of Medicine, and National Taiwan University Hospital, Taipei, Taiwan; 6 Department of Internal Medicine, National Taiwan University College of Medicine, and National Taiwan University Hospital, Taipei, Taiwan; 7 Department of Medical Research, National Taiwan University College of Medicine, and National Taiwan University Hospital, Taipei, Taiwan; 8 Hepatitis Research Center, National Taiwan University College of Medicine, and National Taiwan University Hospital, Taipei, Taiwan; 9 Graduate Institute of Nursing, College of Nursing, Taipei Medical University, Taipei, Taiwan; 10 Sleep Science Center, Taipei Medical University Hospital, Taipei, Taiwan; National Institute of Allergy and Infectious Diseases, United States of America

## Abstract

**Background:**

Interferon-based therapy (IBT) has been the standard of care for hepatitis C virus (HCV) infection. However, conflicting results exist regarding the effects of IBT on risk of developing hepatocellular carcinoma (HCC) and cirrhosis-associated complications, and most included highly selected patients.

**Methods:**

This 8-year cohort study was based on the Longitudinal Health Insurance Database 2000 (LHID 2000) consisting of 1,000,000 beneficiaries randomly selected from all Taiwan National Health Insurance enrollees in 2000 (>23.7 million). Patients with newly detected HCV infections (n = 11,264) were classified based on treatment and clinical outcomes. IBTs were defined as regimens that included interferon- alfa, pegylated interferon- alfa -2a, or pegylated interferon- alfa -2b for at least 3 months. The Cox proportional hazards models were used to estimate the hazard ratio (HR) and associated confidence interval (CI) of HCC and cirrhosis-associated complications for IBT.

**Results:**

The 8-year incidence rate for HCC was 3.9% among patients who received IBT and 5.6% among those who did not. The HCC-free survival rate was significantly higher among patients receiving IBT during the 8-year period than their counterpart (adjusted HR, 0.50; 95% CI, 0.31–0.81; P = .004). Similarly, the event-free survival rates for esophageal variceal bleeding (adjusted HR, 0.45; 95% CI, 0.22–0.91; P = .026), hepatic encephalopathy (adjusted HR, 0.38; 95% CI, 0.21–0.69; P = .001), ascites (adjusted HR, 0.28; 95% CI, 0.14–0.57; P<.001), and cirrhosis (adjusted HR, 0.63; 95% CI, 0.44–0.91; P = .013) were significantly higher among patients who received IBT than those who did not, after adjustment for associated factors.

**Conclusion:**

Treatment with interferon may reduce the 8-year risk of HCC and cirrhosis-associated complications in patients with chronic HCV infection.

## Introduction

Hepatitis C virus (HCV) chronically infects approximately 180 million people worldwide and is one of the major causes of liver disease, including hepatitis, cirrhosis, and hepatocellular carcinoma (HCC) [1], [2]. Although interferon-based therapy (IBT) has been the mainstay of HCV treatment for decades [3]–[5], evidence for its long-term effects on clinical outcomes such as HCC and complications of cirrhosis remains elusive [6]–[10]. Some studies demonstrated beneficial effects of interferon (IFN) for chronic hepatitis C (CHC) patients [11]–[16] whereas others failed to show such effects [17]–[19]. In addition, the effects of IBT among non-responders [9], [20], [21] and those with advanced fibrosis or cirrhosis [22] are inconsistent across different studies. Of note, most published reports that demonstrated decreased risks of HCC or complications of cirrhosis in CHC patients receiving IFN treatment were based on highly selected patients, such as patients who received care at tertiary medical centers [15], [16], [18], [19], [22]–[25], had advanced fibrosis or cirrhosis [7], [12], [14], [18], [22], [26], had liver histological data [11], [19], [23], or failed to previous antiviral treatment [9], or were limited by small sample sizes [7], [14], [18], [19], [22], [26], [27]. The question of whether these observations could be extrapolated to all HCV patients remains unanswered; finding answers to this important clinical question requires large-scale studies.

As Taiwan is a hyperendemic area of chronic viral hepatitis [28], HCC and cirrhosis are the leading causes of death in Taiwanese people [29]. Therefore, the Bureau of Taiwan National Health Insurance (NHI) started to reimburse IBT for CHC patients since 2003. In this study, we analyzed the Longitudinal Health Insurance Database 2000 (LHID 2000) that included a nationally representative population, and used an epidemiologic approach to examine the long-term effects of IBT on risks of HCC and complications of cirrhosis. We hypothesized that IBT is associated with decreased risk of HCC and complications of cirrhosis in chronic hepatitis C patients.

## Patients and Methods

### Ethics Statement

This study was conducted in accordance with the Helsinki Declaration. We used the anonymized LHID2000 data released by Taiwan National Health Research Institutes, which is available to public access for research. Information that could be used to identify patients or care providers, including medical institutions and physicians, was scrambled before being sent to the National Health Research Institutes for database construction and was further scrambled before being released to each researcher. Thus, there is no reasonable basis to believe that the remaining health information could be used to identify a person. All researchers who wish to use the NHIRD and its data subsets are required to sign a written agreement declaring that they have no intention of attempting to obtain information that could potentially violate the privacy of patients or care providers.

### Study Design and Data Source

This study used a retrospective cohort study design. The NHI program in Taiwan is a compulsory program providing universal and quality healthcare to the people at affordable costs. It now covers 99.6% of all the population in Taiwan with an age distribution of 15.65% <15 year-old and 10.74% >65 year-old [30]. The LHID 2000 was released by the Taiwan National Health Research Institutes and includes the original claim data and registration files for 1,000,000 individuals randomly sampled from the 2000 Registry for Beneficiaries of the Taiwan NHI program, which maintains the registration data of any individual who was once a beneficiary of NHI program during the period of 1996–2000. There are approximately 23,720,000 individuals in this registry. The Taiwan National Health Research Institutes claimed that there were no statistically significant differences in gender distribution between the randomly sampled beneficiaries in the LHID 2000 and all beneficiaries under the NHI program.

According to the international consensus recommendations on HCC [31], patients with HCV infection are suggested to receive surveillance for HCC by ultrasonography and a-fetoprotein examinations every 6 months in Taiwan. Moreover, CHC patients receiving IBT are scheduled for follow-up visits and medication refills in at least every 4 weeks. Therefore, in this study, we extracted the following variables of all subjects during their surveillance and follow-up, including HCV diagnosis, patient’s age in years, gender, comorbidities [diabetes mellitus, obesity, human immunodeficiency virus (HIV), alcohol intoxication, ischemic heart diseases, cerebrovascular disease, chronic obstructive pulmonary diseases, chronic renal failure, and hepatitis B (**Table S1**) on the HCV index date], HCC, cirrhosis, hepatic encephalopathy, esophageal varices bleeding, ascites, and the use of IBT.

### Study Sample

The study sample for the current study was patients with the first HCV infection diagnosis between January 1, 2000 and December 31, 2007, identified by using the International Classification of Diseases, Ninth Revision, Clinical Modification (ICD-9-CM) codes (**Table S1**). A total of 12,501 subjects with HCV infection were identified. Subjects with a history of HCV infection before January 1, 2000 (n = 1,237) were excluded, resulting in a total of 11,264 newly detected HCV subjects. Those subjects with unidentified gender (n = 4) were further excluded for the sample used in analyzing each endpoint.

### Interferon-based Treated and Non-treated Cohorts

The independent variable of interest was IBT. IBT was identified by drug codes for interferon alfa, pegylated interferon alfa-2a, pegylated interferon alfa-2b with or without ribavirin (**Table S2**). Because NHI in Taiwan reimbursed CHC patients 4–6 months of interferon or 6 months of pegylated interferon-based treatment during 2000–2008, and most patients who did not achieve early virologic response after 3 months of treatment discontinued IBT [3]–[5], we selected patients receiving ≧3 months of IBT into our analyses, and a cutoff of 6 months to differentiate short and longer-term IBT use. Those patients who maintained IBT for a period of ≧3 months were designated as the treated cohort, and those who received the treatment for a period of <3 months were excluded (n≦55 for analyses of each clinical outcome). The ones who did not receive any IBT between 2000 and 2008 were designated as the control cohort. The treatment cohort was further divided into those who received IBT <6 months and those who received IBT for ≧6 months.

### Study Endpoints and Confounders

The endpoint in this study was whether a HCV patient had a new diagnosis of any one of the selected clinical outcomes, including HCC, cirrhosis, hepatic encephalopathy, esophageal varices bleeding, or ascites. All selected clinical outcomes were identified by using ICD-9-CM codes corresponding to the clinical information during the follow-up. For examples, the diagnosis of HCC was based on either histological confirmation or positive image findings [31]. To confirm a new diagnosis of any one of the selected clinical outcomes and avoid misclassifications of patients who had an early HCC or a milder form of a specific type of event outcomes, all patients with a diagnosis of a specific type of event outcomes before the HCV index date and within 6 months after the HCV index date were excluded before analysis for that specific outcome. All enrolled subjects were tracked until the end of 2008 to identify these clinical outcomes. A combined outcome of cirrhosis, “any cirrhosis complication”, was defined as the presence of any one of the cirrhosis-associated complications, including hepatic encephalopathy, esophageal varices bleeding and ascites.

The survival time was defined as the period between the HCV index date of selection and the first date when a specific event outcome was identified. For those who suffered from multiple episodes of a specific event outcome, only the first event of the respective outcome was included.

We extracted baseline variables frequently associated with HCC and cirrhosis relevant complications on the HCV index date and examined their influences on the impact of IBT and the risk of a specific type of outcomes. These baseline prognostic factors included the patient’s age in years, gender, comorbidities [diabetes mellitus, obesity, human immunodeficiency virus (HIV), alcohol intoxication, ischemic heart diseases, cerebrovascular disease, chronic obstructive pulmonary diseases, chronic renal failure, and hepatitis B] (**Table S1**).

### Statistical Analysis

The Statistical Package for the Social Sciences, version 16.0 (SPSS Inc., Chicago, IL, USA) was used to perform the statistical analyses in this study. The Mann–Whitney U-tests and Chi-square tests were used to examine the differences between treated and non-treated groups. The Cox proportional hazards models were used to estimate the association of IBT with outcomes. We estimated the hazard ratio (HR) and associated confidence interval (CI) for each outcome. In consideration the influences of known prognostic factors on the impact of IBT and the risk of a specific type of outcomes, multivariable Cox models were developed to control for possible confounding variables. To examine the effects of different IBT durations on the risk of HCC and cirrhosis-associated complications, the risk of HCC and cirrhosis-associated complications in patients who received <6 months of IBT and patients who received ≧6 months of IBT was compared with that of those who were not treated. Interaction terms for IBT and each of the confounders were included in separate models. The loss to follow-up rate (withdrawing from the NHI Program) was calculated between IBT treated and non-treated groups for all endpoints analyzed. The proportional hazards assumption was tested and was not violated.

We also calculated the number of patients needed to be treated for one additional patient to benefit (number needed to benefit, NNTB) or be harmed (number needed to harm, NNTH) [32].

As the occurrence of cirrhosis might impact the prognosis of HCV patients, we further extracted the number of patients with a diagnosis of cirrhosis before the IBT was initiated, and examined the effect of IBT on outcomes by Cox proportional hazards models and adjusted for cirrhosis occurring after the index day (i.e., between the HCV index date and the identification of outcomes or till the end of 2008 for those without outcomes).

Because the IBT group might receive a closer check-up than the non-IBT group, we further compared the number of ultrasonography and alpha-fetoprotein (AFP) examinations between IBT and non-IBT groups to examine this inherited “detection bias” introduced by the differential surveillance and follow-up between groups. The number of ultrasonography examinations was further adjusted in Cox proportional hazards models testing the effects of IBT on endpoints of interest.

## Results

### Demographic Data

Between January 1, 2000 and December 31, 2007, 11,264 patients with a newly diagnosis of HCV infection were identified. Finally, 10,058 without a diagnosis of HCC, 10,768 without a diagnosis of esophageal varices bleeding, 10,762 without a diagnosis of hepatic encephalopathy, 10,642 without a diagnosis of ascites, 8,964 without a diagnosis of cirrhosis, and 10,366 without a diagnosis of “any cirrhosis complication” before the HCV index date and within 6 months after the HCV index date were included for analyses of the association of IBT with each clinical outcome. The IBT usage rates between the included and excluded HCV patients were comparable for each clinical outcome, while the loss to follow-up rate (withdrawing from the NHI Program) was significantly higher in the non-IBT treated groups for all endpoints analyzed (hepatocellular carcinoma [**Table S3-a**], esophageal varices bleeding [**Table S3-b**], hepatic encephalopathy, [**Table S3-c**],ascites [**Table S3-d**], cirrhosis [**Table S3-e**], and any cirrhosis complication [**Table S3-f**]; All P<0.001).

The overall person-years of follow-up for different clinical outcomes–HCC, esophageal varices bleeding, hepatic encephalopathy, ascites, cirrhosis, and “any cirrhosis complication” were 48217.4, 52671.4, 52294.0, 51635.9, 41724.7, and 49754.4, respectively. The median duration of follow-up was 4.7, 4.8, 4.7, 4.7, 4.5, and 4.6 years, respectively, for HCC, esophageal varices bleeding, hepatic encephalopathy, ascites, cirrhosis, and “any cirrhosis complication”. The information on the person-years of follow-up for other clinical outcomes stratified by treatment group (i.e., no treatment, <6 months of IBT use, and ≧6 months of IBT use) and the median follow-up duration are provided in **Tables S4 and S5**. Of those who received IBT, approximately 98.4% received pegylated interferon alfa-2b combined with ribavirin. The median duration of treatment was 6 months (inter-quartile range: 5–6 months). The distributions of selected demographic characters were comparable between the IBT treated and non-treated cohorts, with the exception that the treated cohort was male predominant, younger, and had fewer HBV infection and fewer chronic renal failure than those in the non-treated cohort (**Table 1**). Moreover, the mean number of ultrasonography and AFP examinations were significantly higher in the IBT group compared with the non-treated group for all endpoints (hepatocellular carcinoma [**Table S6-a**], esophageal varices bleeding [**Table S6-b**], hepatic encephalopathy, [**Table S6-c**], ascites [**Table S6-d**], cirrhosis [**Table S6-e**], and any cirrhosis complication [**Table S6-f**]; All P<0.001).

**Table 1:** Baseline characteristics of Hepatitis C patients enrolled for analyses of clinical outcomes, including hepatocellular carcinoma and complications of cirrhosis, stratified according to the use of interferon.

	Hepatocellular Carcinoma, n = 10,058	
	Use of IBT	
	Yes (n = 457)	No (n = 9,601)
Age, yrs Median (25th–75th)	50 (42–57)	53 (41–65)^a^
Male, n (%)	264 (57.8)	4836 (50.4)^a^
DM, n (%)	34 (7.4)	872 (9.1)
Obesity, n (%)	0 (0.0)	11 (0.1)
HIV, n (%)	2 (0.4)	36 (0.4)
Alcohol intoxication, n (%)	1 (0.2)	29 (0.3)
IHD, n (%)	2 (0.4)	148 (1.5)
CVD, n (%)	1 (0.2)	108 (1.1)
COPD, n (%)	6 (1.3)	228 (2.4)
Hepatitis B, n (%)	28 (6.1)	1540 (16)^a^
CRF, n (%)	1 (0.2)	205 (2.1)^a^
	**Esophageal varices bleeding, n = 10,768**	
	**Use of IBT**	
	**Yes (n = 515)**	**No (n = 10,253)**
Age, yrs Median (25th –75th)	50 (42–58)	54 (42–66)^a^
Male, n (%)	301 (58.4)	5226 (51.0)^a^
DM, n (%)	37 (7.2)	949 (9.3)
Obesity, n (%)	0 (0.0)	11 (0.1)
HIV, n (%)	2 (0.4)	36 (0.4)
Alcohol intoxication, n (%)	1 (0.2)	27 (0.3)
IHD, n (%)	2 (0.4)	160 (1.6)
CVD, n (%)	2 (0.4)	115 (1.1)
COPD, n (%)	6 (1.2)	243 (2.4)
Hepatitis B, n (%)	34 (6.6)	1649 (16.1) a
CRF, n (%)	1 (0.2)	223 (2.2)^a^
	**Hepatic encephalopathy, n = 10,762**	
	**Use of IBT**	
	**Yes (n = 518)**	**No (n = 10,244)**
Age, yrs Median (25th –75th)	50 (43–58)	54 (42–66)^a^
Male, n (%)	303 (58.5)	5229 (51.0)^a^
DM, n (%)	38 (7.3)	949 (9.3)
Obesity, n (%)	0 (0.0)	11 (0.1)
HIV, n (%)	2 (0.4)	36 (0.4)
Alcohol intoxication, n (%)	1 (0.2)	29 (0.3)
IHD, n (%)	2 (0.4)	160 (1.6)
CVD, n (%)	2 (0.4)	111 (1.1)
COPD, n (%)	6 (1.2)	240 (2.3)
Hepatitis B, n (%)	35 (6.8)	1641 (16)^a^
CRF, n (%)	1 (0.2)	220 (2.1)^a^
	**Ascites, n = 10,642**	
	**Use of IBT**	
	**Yes (n = 516)**	**No (n = 10,126)**
Age, yrs Median (25th –75th)	50 (42.–58)	54 (42–66)^a^
Male, n (%)	305 (59.1)	5161 (51.0)^a^
DM, n (%)	39 (7.6)	933 (9.2)
Obesity, n (%)	0 (0.0)	11 (0.1)
HIV, n (%)	2 (0.4)	36 (0.4)
Alcohol intoxication, n (%)	1 (0.2)	27 (0.3)
IHD, n (%)	2 (0.4)	158 (1.6)
CVD, n (%)	2 (0.4)	114 (1.1)
COPD, n (%)	6 (1.2)	240 (2.4)
Hepatitis B, n (%)	35 (6.8)	1631 (16.1) a
CRF, n (%)	1 (0.2)	209 (2.1)^a^
	**Cirrhosis, n = 8,964**	
	**Use of IBT**	
	**Yes (n = 373)**	**No (n = 8,591)**
Age, yrs Median (25th –75th)	49 (40–56)	52 (40–64)^a^
Male, n (%)	219 (58.7)	4295 (50.0)^a^
DM, n (%)	31 (8.3)	744 (8.7)
Obesity, n (%)	0 (0.0)	11 (0.1)
HIV, n (%)	2 (0.5)	35 (0.4)
Alcohol intoxication, n (%)	1 (0.3)	15 (0.2)
IHD, n (%)	2 (0.5)	131 (1.5)
CVD, n (%)	2 (0.5)	92 (1.1)
COPD, n (%)	5 (1.3)	195 (2.3)
Hepatitis B, n (%)	21 (5.6)	1403 (16.3)^a^
CRF, n (%)	1 (0.3)	166 (1.9)^a^
	**Any cirrhosis complication, n = 10,366**	
	**Use of IBT**	
	**Yes (n = 509)**	**No (n = 9,857)**
Age, yrs Median (25th–75th)	50 (42–58)	53 (41–65)^a^
Male, n (%)	299 (58.7)	5003 (50.8)^a^
DM, n (%)	37 (7.3)	895 (9.1)
Obesity, n (%)	0 (0.0)	11 (0.1)
HIV, n (%)	2 (0.4)	36 (0.4)
Alcohol intoxication, n (%)	1 (0.2)	24 (0.2)
IHD, n (%)	2 (0.4)	157 (1.6)^a^
CVD, n (%)	2 (0.4)	108 (1.1)
COPD, n (%)	6 (1.2)	233 (2.4)
Hepatitis B, n (%)	34 (6.7)	1592 (16.2)^a^
CRF, n (%)	1 (0.2)	201 (2.0)^a^

a P<0.05. Tested by the Mann-Whitney U test and the Chi-square test. Sample sizes for each of the clinical outcomes indicate the number of subjects WITHOUT the diagnosis of that clinical outcome before and within 6 months after the HCV index date. **Abbreviations:** IBT, interferon-based therapy; DM, diabetes mellitus; HIV, human immunodeficiency virus; IHD, ischemic heart diseases; CVD, Cerebrovascular disease; COPD, chronic obstructive pulmonary diseases; CRF, chronic renal failure.

### Impact of IBT on the Risk of HCC, Cirrhosis, Hepatic Encephalopathy, Esophageal Varices Bleeding, and Ascites

The 8-year incidence of HCC for the IBT-treated and non-treated groups was 3.9% and 5.6%, respectively. Among the IBT-treated patients, the incidence of HCC was 3.1% and 4.9%, respectively, for those who received IBT for 6 months and longer and those who received IBT for less than 6 months. The overall incidence of other clinical outcomes and the incidence stratified by treatment group were presented in **Table S7**. The NNTB for these clinical outcomes were 59 for HCC, 159 for esophageal variceal bleeding, 70 for hepatic encephalopathy, 48 for ascites, 94 for cirrhosis, and 42 for “any cirrhosis complication” (Data not shown, please see **Table S8**).

Patients in the treated cohort had a higher survival rate for all newly detected clinical outcomes than the ones in non-treated cohort, and the differences were significant for ascites (p = 0.013) and “any cirrhosis complication” (p = 0.016) but not for HCC, cirrhosis, esophageal varices bleeding and hepatic encephalopathy (Unadjusted Model, **Table 2**).

**Table 2:** The effect of interferon-based therapy on risks of hepatocellular carcinoma and cirrhosis complications.

		HCC (n = 10,058)	Esophageal variceal bleeding (n = 10,768)	Hepatic encephalopathy (n = 10,762)	Ascites (n = 10,642)	Cirrhosis (n = 8,964)		Any cirrhosis complication (n = 10,366)
IBT		HR (95% CI)						
Unadjusted Model	Yes	0.68 (0.42–1.09)	0.70 (0.35–1.41)	0.58 (0.32–1.06)	0.41 (0.20–0.83)^a^		0.87 (0.60–1.25)	0.53 (0.32–0.89)^a^
	No	1	1	1	1		1	1
Adjusted Model 1	Yes	0.53 (0.33–0.85)^*a^	0.49 (0.24–1.00)^*^	0.40 (0.22–0.73)^*a^	0.27 (0.14–0.55)^*a^		–	0.33 (0.20–0.54)^*a^
	No	1	1	1	1		1	1
Adjusted Model 2	Yes	0.51 (0.32–0.82)^†a^	0.43 (0.21–0.88)^†a^	0.37 (0.20–0.67)^†a^	0.27 (0.13–0.55)^†a^		0.63 (0.43–0.91)^§a^	0.34 (0.20–0.57)^#a^
	No	1	1	1	1		1	1
Adjusted Model 3	Yes	0.50 (0.31–0.81)^‡a^	0.45 (0.22–0.91)^‡a^	0.38 (0.21–0.69)^‡a^	0.28 (0.14–0.57)^‡a^		0.63 (0.44–0.91)^£a^	0.35 (0.21–0.59)^‡a^
	No	1	1	1	1		1	1

a P<0.05. Tested by Cox regression. Sample sizes for each of the clinical outcomes indicate the number of subjects WITHOUT the diagnosis of that clinical outcome before and within 6 months after the HCV index date. ^*^ Adjusted for cirrhosis after the index day. ^†^ Adjusted for age, sex, hepatitis B, CRF, cirrhosis after the index day, and the number of ultrasonography examinations after the index day. ^#^Adjusted age, sex, IHD, hepatitis B, CRF, cirrhosis after the index day, and the number of ultrasonography examinations after the index day. ^‡^ Adjusted for age, sex, diabetes mellitus, IHD, cerebrovascular disease, COPD, hepatitis B, CRF, cirrhosis after the index day, and the number of ultrasonography examinations after the index day. ^§^Adjusted for age, sex, hepatitis B, CRF, and the number of ultrasonography examinations after the index day. ^£^Adjusted for age, sex, diabetes mellitus, IHD, cerebrovascular disease, COPD, hepatitis B, CRF, and the number of ultrasonography examinations after the index day. Abbreviations: IBT, interferon-based therapy; HCC, hepatocellular carcinoma; IHD, ischemic heart diseases; COPD, chronic obstructive pulmonary diseases; CRF, chronic renal failure.

The percentage of HCV patients with a diagnosis of cirrhosis before the IBT for patients who developed different endpoints–HCC, esophageal varices bleeding, hepatic encephalopathy, ascites and “any cirrhosis complication “ was 22.8%, 27%, 27.4%, 27.5%, and 26.5%, respectively. IBT significantly increased the HCC-free survival rate (adjusted HR = 0.53, 95% CI = 0.33–0.85, p = 0.008), decreased the risk of hepatic encephalopathy (adjusted HR = 0.40, 95% CI = 0.22–0.73, p = 0.003), ascites (adjusted HR = 0.27, 95% CI = 0.14–0.55, p<0.001), and “any cirrhosis complication” (adjusted HR = 0.33, 95%CI = 0.20–0.54, p<0.001) after adjusting for cirrhosis occurring after the index day. However, the two groups were not significantly different in the survival rate for esophageal varices bleeding. After adjusting for factors that significantly differed between groups, IBT had a lower risk of HCC (adjusted HR, 0.51; 95% CI, 0.32–0.82), esophageal varices bleeding (adjusted HR, 0.43; 95% CI, 0.21–0.88), hepatic encephalopathy (adjusted HR, 0.37; 95% CI, 0.20–0.67), ascites (adjusted HR, 0.27; 95% CI, 0.13–0.55), cirrhosis (adjusted HR, 0.63; 95% CI, 0.43–0.91) and “any cirrhosis complication” (adjusted HR, 0.34; 95% CI, 0.20–0.57) (Adjusted Models 2 in **Table 2**). The results remained largely unchanged after adjusting all confounders in the multivariable Cox models (Adjusted Models 3 in **Table 2**). As the prevalence of obesity, HIV, and alcohol intoxication was extremely low (see **Table 1**), we did not include these variables in the models. No significant interactions were found between IBT and each of the confounders.

For sensitivity analyses, we excluded subjects coinfected with HBV and HIV from the multivariable Cox proportional hazard model testing the effect of IBT on the risk of HCC. The adjusted HR of HCC was 0.50 (95% CI, 0.31–0.81, p = 0.005) for the IBT-treated group as compared with the non-treated group after adjusting for age, sex, diabetes mellitus, ischemic heart diseases, cerebrovascular disease, COPD, chronic renal failure, cirrhosis after the index day, and number of ultrasonography examinations after the index day.

### Impact of IBT Duration on the Risk of HCC, Cirrhosis, Hepatic Encephalopathy, Esophageal Varices Bleeding, and Ascites

**Figure 1:**
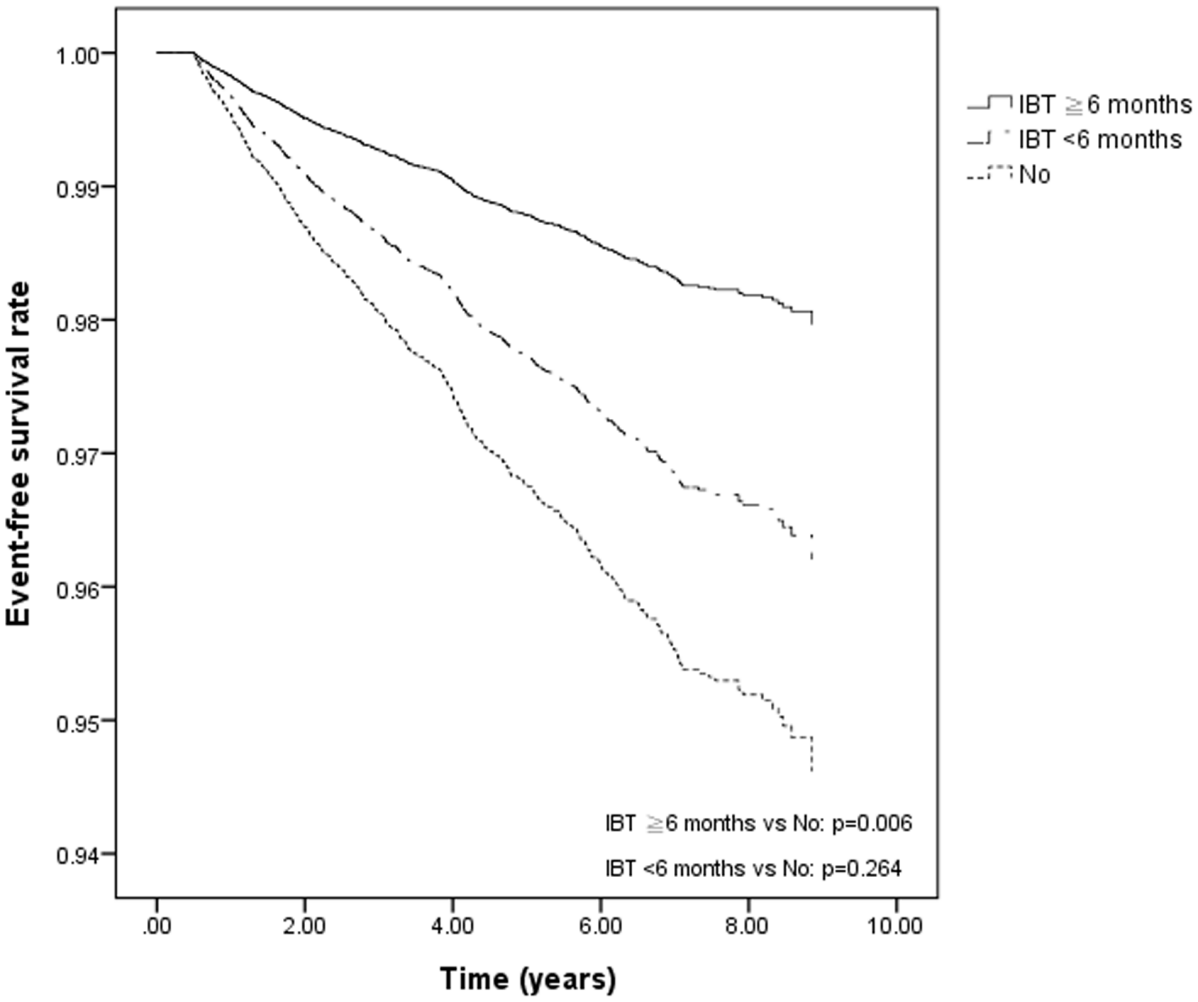
Hepatocellular carcinoma-free survival rate by different interferon-based treatment conditions as estimated by the Cox proportional hazards model in patients with hepatitis C viral infection. <6 months IBT *vs.* no treatment, p = 0.264; ≧6 months IBT *vs.* no treatment, p = 0.006. IBT = interferon-based treatment. The y-axis is modified, so that it only displays the survival estimates between 0.94 and 1.00.

Compared to those who were not treated, patients who received ≧6 months of IBT had a lower risk of HCC (HR, 0.40; 95% CI, 0.20–0.80), hepatic encephalopathy (HR, 0.24; 95% CI, 0.09–0.64), ascites (HR, 0.27; 95% CI, 0.11–0.66), and “any cirrhosis complication” (HR, 0.32; 95% CI, 0.16–0.61) after adjusting for cirrhosis occurring after the index day. However, a decreased risk was only observed for ascites and “any cirrhosis complication” in patients who received <6 months of IBT compared with those who were not treated (**Table 3**, Adjusted Model 1). The differences in HCC-free survival, hepatic encephalopathy-free survival, ascites-free survival, cirrhosis-free survival, and any cirrhosis complication-free survival between patients who received ≧6 months of IBT and non-treated ones were statistically significant after further adjustment of factors that differed significantly between two groups (Adjusted Model 2 in **Table 3** **and** **Figure 1**). After adjusting for all confounders, similar results were found (Adjusted Model 3 in **Table 3**). No significant interactions were found between IBT and each of the confounders. Of particular note, the prevalence of obesity, HIV, and alcohol intoxication was extremely low; we therefore did not include these variables in the models. In adjusted Model 2 and Model 3, a decreased risk was only observed for ascites and “any cirrhosis complication” in patients who received <6 months of IBT compared with those who were not treated (**Table 3**).

**Table 3:** The effect of duration of interferon-based therapy on risks of hepatocellular carcinoma and cirrhosis complications.

		HCC (n = 10,058)	Esophageal variceal bleeding (n = 10,768)	Hepatic encephalopathy (n = 10,762)	Ascites (n = 10,642)	Cirrhosis (n = 8,964)	Any cirrhosis complication (n = 10,366)
IBT		HR (95% CI)					
Unadjusted Model	≧6 months	0.55 (0.28–1.11)	0.94 (0.42–2.11)	0.38 (0.14–1.02)	0.46 (0.19–1.11)	0.86 (0.52–1.41)	0.57 (0.30–1.11)
	<6 months	0.83 (0.44–1.55)	0.39 (0.10–1.59)	0.84 (0.40–1.77)	0.35 (0.11–1.09)	0.88 (0.52–1.49)	0.48 (0.22–1.08)
	No	1	1	1	1	1	1
Adjusted Model 1	≧6 months	0.40 (0.20–0.80)^*a^	0.61 (0.27–1.36)^*^	0.24 (0.09–0.64)^*a^	0.27 (0.11–0.66)^*a^		0.32 (0.16–0.61)^*a^
	<6 months	0.71 (0.38–1.34)^*^	0.32 (0.08–1.28)^*^	0.65 (0.31–1.38)^*^	0.27 (0.09–0.83)^*a^		0.34 (0.15–0.76)^*a^
	No	1	1	1	1	1	1
Adjusted Model 2	≧6 months	0.38 (0.19–0.76)^†a^	0.50 (0.22–1.13)^†^	0.21 (0.08–0.57)^†a^	0.27 (0.11–0.65)^†a^	0.57 (0.35–0.94)^§a^	0.32 (0.17–0.63)^†a^
	<6 months	0.71 (0.38–1.32) ^†^	0.31 (0.08–1.24)^†^	0.63 (0.30–1.33)^†^	0.28 (0.09–0.86)^†a^	0.69 (0.41–1.18)^§^	0.37 (0.16–0.82)^†a^
	No	1	1	1	1	1	1
Adjusted Model 3	≧6 months	0.37 (0.19–0.75)^‡a^	0.52 (0.23–1.17)^‡^	0.22 (0.08–0.59)^‡a^	0.28 (0.12–0.68)^‡a^	0.58 (0.35–0.95)^£a^	0.34 (0.17–0.66)^‡a^
	<6 months	0.70 (0.37–1.31)^‡^	0.32 (0.08–1.28)^‡^	0.64 (0.30–1.36)^‡^	0.29 (0.09–0.89)^‡a^	0.70 (0.41–1.19)^£^	0.38 (0.17–0.85)^‡a^
	No	1	1	1	1	1	1

a P<0.05. Tested by Cox regression. Sample sizes for each of the clinical outcomes indicate the number of subjects WITHOUT the diagnosis of that clinical outcome before and within 6 months after the HCV index date. ^*^ Adjusted for cirrhosis after the index day. ^†^ Adjusted for age, sex, hepatitis B, CRF, cirrhosis after the index day, and the number of ultrasonography examinations after the index day. ^‡^ Adjusted for age, sex, diabetes mellitus, IHD, cerebrovascular disease, COPD, hepatitis B, CRF, cirrhosis after the index day, and the number of ultrasonography examinations after the index day. ^§^Adjusted for age, sex, hepatitis B, and the number of ultrasonography examinations after the index day. ^£^Adjusted for age, sex, diabetes mellitus, IHD, cerebrovascular disease, COPD, hepatitis B, CRF, and the number of ultrasonography examinations after the index day. **Abbreviations:** IBT, interferon-based therapy; HCC, hepatocellular carcinoma; IHD, ischemic heart diseases; COPD, chronic obstructive pulmonary diseases; CRF, chronic renal failure.

## Discussion

As the progression of chronic HCV infection is slow, the current goal of HCV therapy is defined by a surrogate virological parameter, sustained virologic response (SVR), rather than a clinical endpoint, such as HCC or complications of cirrhosis. SVR is usually considered synonymous with cure and associated with decreasing cirrhosis complications [16], [26], [33], [34], though the data supporting this assumption remain inconclusive. To date, no randomized, controlled trials of IBT have demonstrated a beneficial impact on overall mortality, liver specific mortality or development of HCC, and most of the evidence in favor of IBT is inferred from data of highly selected patients, clinical trials, or cirrhotic patients in tertiary medical centers [7], [9], [11], [12], [14]–[16], [18], [19], [22]–[24], [26], [33], [35]. Therefore, a study based on general population, a large sample size, and long-term observations for complications of cirrhosis is necessary to confirm this assumption. To the best of our knowledge, our study is by far the largest general population-based study that provides evidence to rationalize the use of IBT for patients with HCV infection. Our data clearly demonstrated beneficial effects of IBT on preventing HCC as we found that the HR after adjusting for known prognostic factors in the treated cohort relative to the untreated cohort was 0.5. Similarly, IBT was associated with a 55%, 62%, 72%, 37%, and 65% reduction of risk for esophageal varices bleeding, hepatic encephalopathy, ascites, cirrhosis, and any cirrhosis-associated complication, respectively, after adjustment for prognostic factors.

Our data not only revealed beneficial effects of IBT on preventing HCC, esophageal varices bleeding, hepatic encephalopathy, ascites, cirrhosis, or any cirrhosis-associated complication during an 8-year observation, but also demonstrated that longer treatment duration (at least 6 months) may be required for IBT to exert its protecting effects on HCC, hepatic encephalopathy, and cirrhosis. Moreover, our data provided evidence for the development of clinical practice guidelines as we found that the average number of HCV patients needed to be treated with IBT for 3 months or more for one additional patient to benefit or be harmed. Nonetheless, it must be kept in mind that deteriorations in health-related quality of life and adverse effects of IBT, both are major concerns in current HCV management [36] and important issues in justifying the treatment, were not evaluated in this study. Moreover, whether IBT is cost-effective remains unclear and awaits further studies. Finally, oral antiviral agents for the treatment of HCV have been demonstrated to improve virological responses of patients with chronic HCV infection and are likely to be available in recent years. However, there remains no available study examining the long-term effects of oral antiviral agents on HCV patients, and future studies exploring this important issue are needed.

This study has all the limitations of retrospective cohort studies in attributing causality. First, this study analyzed *ex post facto* patients groups, and hence certain selection biases may exist in our study sample. For examples, during the period from 2000 to 2008, the Taiwan NHI program only reimbursed IBT for CHC patients who had a histologic confirmation of chronic hepatitis, at least mild fibrosis, and serum alanine aminotransferase (ALT) levels at least twice the upper limit of normal on two occasions within 6 months before the initiation of treatment. Second, pretreatment data on biochemical, virologic response, and histological characteristics were generally lacking in the LHID 2000, and thus were not accounted for when examining the effects of IFN on complications of cirrhosis. Although previous meta-analyses have demonstrated an anti-fibrotic effect of interferon in patients who have not sustained biochemical and virologic responses [37], this conclusion is challenged by recent studies focused on HCV/HIV [38] and advanced hepatitis C nonresponders [10]. Therefore, future studies examining the long-term effects of IBT on patients with different HCV genotypes infection, milder liver fibrosis, non-responders, replapsers, or HIV/HCV infection are needed. Third, although our study demonstrated decreased risks of clinical outcomes in patients receiving ≧3 months of treatments and implied that 6 months of IBT treatment might be required to achieve effects for such clinical outcomes as HCC, encephalopathy, and cirrhosis, the question of whether more than 12 months of IBT would be beneficial for CHC patients remains unclear. Fourth, elderly patients have increasing risks of HCC and advanced fibrosis [39], and several prognostic factors, such sex, diabetes, obesity, and cirrhosis were likely to confound the results of this study. Nevertheless, known prognostic factors were adjusted for in our analyses and IBT was identified as an independent protective factor. Moreover, although Taiwan is endemic in HBV infection and the prevalence of HBV and HIV may impact the outcomes of treatment, excluding subjects co-infected with HBV and HIV did not dramatically change the results. Fifth, because the loss to follow-up rate was significantly higher in the non-IBT treated groups for all endpoints analyzed, the incidence of the endpoints in the non-IBT treated group might have been underestimated due to the fact that death is the major cause of loss to follow-up. However, if this was the case it would work in favor of our hypothesis. Moreover, as the IBT group was indeed under a closer check-up than the non-IBT group, this detection bias might have potentially underestimated the incidence of outcomes in the non-IBT group which in turn potentially underestimated the beneficial effect of IBT; or alternatively, it might have leaded to better health care and behavior in the IBT group and consequently might overestimate the observed beneficial effect of IBT on CHC patients. Therefore, we have examined the number of ultrasonography examinations after the index day, and found patients in the treated cohort received more frequent ultrasonography examinations than those in non-treated cohort (p<0.05). However, adding the number of ultrasonography examinations after the index day into all multivariate models, the conclusions showed that IBT had a lower risk of HCC and cirrhosis-associated complications. Moreover, comparing to non-treated patients, patients receiving IBT ≥6 months have a significantly lower risk of cirrhosis-associated complications and HCC, with the exception of esophageal varices bleeding. Last, the confirmation of HCC and development of liver outcomes were based on claims data, and most patients had a clinical diagnosis based on their image studies or laboratory data, not a histologic diagnosis. Thus the incidence of each outcome might have been underestimated. Moreover, misclassification and measurement errors in drug exposure might have occurred if the patients failed to take the prescribed drugs. Non-compliance is also likely to cause underestimation of the drug effect. Nonetheless, the use of a large nationally representative sample, the dramatically reduced HCC and cirrhosis-associated risk, and the fact that data were collected originally for a different purpose increase the likelihood of the findings being valid, and indicate that these results are likely to be applicable to the whole population and across the different spectrum of CHC patients who meet the criteria for IBT treatment.

In conclusion, our nationwide long-term cohort study demonstrated that IBT was associated with a 50% reduction of risk of HCC and 37% to 72% reductions of risk for complications of cirrhosis in chronic hepatitis C patients, independent of other confounders. Our results may provide evidence to justify the use of IBT in chronic hepatitis C patients and to support the long-term benefits of IBT in reducing the risk of liver cancer and cirrhosis-associated complications.

## Supporting Information

Table S1 International Classification of Diseases, Ninth Revision, Clinical Modification (ICD-9-CM) codes used in the study.
(DOC)

Table S2 Interferon-based regimens for treatment of hepatitis C viral infection in Taiwan.
(DOC)

Table S3 Distribution of the loss to follow-up rate for each clinical outcome.
(DOC)

Table S4 Total person-years of follow-up for each of the clinical outcomes stratified by treatment group.
(DOC)

Table S5 Median years of follow-up for each of the clinical outcomes according to treatment group.
(DOC)

Table S6 The number of ultrasonography and a-fetoprotein examinations between IBT treated and non-treated cohorts for each clinical outcome.
(DOC)

Table S7 Incidence of clinical outcomes according to treatment group.
(DOC)

Table S8 The number needed to treat for each clinical outcome.
(DOC)

## References

[1] Kao JH, Chen DS (2000) Transmission of hepatitis C virus in Asia: past and present perspectives. J Gastroenterol Hepatol 15 Suppl: E91–96 10.1046/j.1440-1746.2000.02108.x 10921389

[2] Chen DS (1995) Hepatitis C virus in chronic liver disease and hepatocellular carcinoma in Taiwan. Princess Takamatsu Symp 25: 27–32. 8875606

[3] Ghany MG, Strader DB, Thomas DL, Seeff LB (2009) Diagnosis, management, and treatment of hepatitis C: an update. Hepatology 49: 1335–1374. 19330875 10.1002/hep.22759 PMC7477893

[4] European Association for the Study of the Liver (2011) EASL Clinical Practice Guidelines: management of hepatitis C virus infection. J Hepatol 55: 245–264. 21371579 10.1016/j.jhep.2011.02.023

[5] McCaughan GW, Omata M, Amarapurkar D, Bowden S, Chow WC, et al (2007) Asian Pacific Association for the Study of the Liver consensus statements on the diagnosis, management and treatment of hepatitis C virus infection. J Gastroenterol Hepatol 22: 615–633. 17444847 10.1111/j.1440-1746.2007.04883.x

[6] Okanoue T, Minami M, Makiyama A, Sumida Y, Yasui K, et al (2005) Natural course of asymptomatic hepatitis C virus-infected patients and hepatocellular carcinoma after interferon therapy. Clin Gastroenterol Hepatol 3: S89–91. 16234069 10.1016/s1542-3565(05)00701-9

[7] Gramenzi A, Andreone P, Fiorino S, Camma C, Giunta M, et al (2001) Impact of interferon therapy on the natural history of hepatitis C virus related cirrhosis. Gut 48: 843–848. 11358906 10.1136/gut.48.6.843 PMC1728334

[8] Shiffman ML, Morishima C, Dienstag JL, Lindsay KL, Hoefs JC, et al (2009) Effect of HCV RNA suppression during peginterferon alfa-2a maintenance therapy on clinical outcomes in the HALT-C trial. Gastroenterology 137: 1986–1994. 19747918 10.1053/j.gastro.2009.08.067 PMC3774149

[9] Di Bisceglie AM, Shiffman ML, Everson GT, Lindsay KL, Everhart JE, et al (2008) Prolonged therapy of advanced chronic hepatitis C with low-dose peginterferon. N Engl J Med 359: 2429–2441. 19052125 10.1056/NEJMoa0707615 PMC2606037

[10] Lok AS, Everhart JE, Wright EC, Di Bisceglie AM, Kim HY, et al. (2011) Maintenance peginterferon therapy and other factors associated with hepatocellular carcinoma in patients with advanced hepatitis C. Gastroenterology 140: 840–849; quiz e812. 10.1053/j.gastro.2010.11.050 PMC3057272 21129375

[11] Yoshida H, Shiratori Y, Moriyama M, Arakawa Y, Ide T, et al (1999) Interferon therapy reduces the risk for hepatocellular carcinoma: national surveillance program of cirrhotic and noncirrhotic patients with chronic hepatitis C in Japan. IHIT Study Group. Inhibition of Hepatocarcinogenesis by Interferon Therapy. Ann Intern Med 131: 174–181. 10428733 10.7326/0003-4819-131-3-199908030-00003

[12] Mazzella G, Accogli E, Sottili S, Festi D, Orsini M, et al (1996) Alpha interferon treatment may prevent hepatocellular carcinoma in HCV-related liver cirrhosis. J Hepatol 24: 141–147. 8907566 10.1016/s0168-8278(96)80022-5

[13] Imai Y, Kawata S, Tamura S, Yabuuchi I, Noda S, et al (1998) Relation of interferon therapy and hepatocellular carcinoma in patients with chronic hepatitis C. Osaka Hepatocellular Carcinoma Prevention Study Group. Ann Intern Med 129: 94–99. 9669992 10.7326/0003-4819-129-2-199807150-00005

[14] Benvegnu L, Chemello L, Noventa F, Fattovich G, Pontisso P, et al (1998) Retrospective analysis of the effect of interferon therapy on the clinical outcome of patients with viral cirrhosis. Cancer 83: 901–909. 9731893 10.1002/(sici)1097-0142(19980901)83:5<901::aid-cncr15>3.0.co;2-z

[15] Veldt BJ, Heathcote EJ, Wedemeyer H, Reichen J, Hofmann WP, et al (2007) Sustained virologic response and clinical outcomes in patients with chronic hepatitis C and advanced fibrosis. Ann Intern Med 147: 677–684. 18025443 10.7326/0003-4819-147-10-200711200-00003

[16] Manesis EK, Papatheodoridis GV, Touloumi G, Karafoulidou A, Ketikoglou J, et al (2009) Natural course of treated and untreated chronic HCV infection: results of the nationwide Hepnet.Greece cohort study. Aliment Pharmacol Ther 29: 1121–1130. 19222410 10.1111/j.1365-2036.2009.03974.x

[17] Niederau C, Lange S, Heintges T, Erhardt A, Buschkamp M, et al (1998) Prognosis of chronic hepatitis C: results of a large, prospective cohort study. Hepatology 28: 1687–1695. 9828236 10.1002/hep.510280632

[18] Fattovich G, Giustina G, Degos F, Diodati G, Tremolada F, et al (1997) Effectiveness of interferon alfa on incidence of hepatocellular carcinoma and decompensation in cirrhosis type C. European Concerted Action on Viral Hepatitis (EUROHEP). J Hepatol 27: 201–205. 9252096 10.1016/s0168-8278(97)80302-9

[19] Aizawa Y, Shibamoto Y, Takagi I, Zeniya M, Toda G (2000) Analysis of factors affecting the appearance of hepatocellular carcinoma in patients with chronic hepatitis C. A long term follow-up study after histologic diagnosis. Cancer 89: 53–59. 10897000

[20] Papatheodoridis GV, Papadimitropoulos VC, Hadziyannis SJ (2001) Effect of interferon therapy on the development of hepatocellular carcinoma in patients with hepatitis C virus-related cirrhosis: a meta-analysis. Aliment Pharmacol Ther 15: 689–698. 11328263 10.1046/j.1365-2036.2001.00979.x

[21] Camma C, Di Bona D, Schepis F, Heathcote EJ, Zeuzem S, et al (2004) Effect of peginterferon alfa-2a on liver histology in chronic hepatitis C: a meta-analysis of individual patient data. Hepatology 39: 333–342. 14767986 10.1002/hep.20073

[22] Hu KQ, Tong MJ (1999) The long-term outcomes of patients with compensated hepatitis C virus-related cirrhosis and history of parenteral exposure in the United States. Hepatology 29: 1311–1316. 10094980 10.1002/hep.510290424

[23] Ikeda K, Saitoh S, Arase Y, Chayama K, Suzuki Y, et al (1999) Effect of interferon therapy on hepatocellular carcinogenesis in patients with chronic hepatitis type C: A long-term observation study of 1,643 patients using statistical bias correction with proportional hazard analysis. Hepatology 29: 1124–1130. 10094956 10.1002/hep.510290439

[24] Yu ML, Lin SM, Chuang WL, Dai CY, Wang JH, et al (2006) A sustained virological response to interferon or interferon/ribavirin reduces hepatocellular carcinoma and improves survival in chronic hepatitis C: a nationwide, multicentre study in Taiwan. Antiviral therapy 11: 985–994. 17302368

[25] Yu ML, Dai CY, Chen SC, Lee LP, Hsieh MY, et al (2005) High versus standard doses interferon-alpha in the treatment of naive chronic hepatitis C patients in Taiwan: a 10-year cohort study. BMC Infect Dis 5: 27. 15823212 10.1186/1471-2334-5-27 PMC1090579

[26] Cardoso AC, Moucari R, Figueiredo-Mendes C, Ripault MP, Giuily N, et al (2010) Impact of peginterferon and ribavirin therapy on hepatocellular carcinoma: incidence and survival in hepatitis C patients with advanced fibrosis. J Hepatol 52: 652–657. 20346533 10.1016/j.jhep.2009.12.028

[27] Hung CH, Lu SN, Wang JH, Hu TH, Chen CH, et al (2011) Sustained HCV clearance by interferon-based therapy reduces hepatocellular carcinoma in hepatitis B and C dually-infected patients. Antiviral therapy 16: 959–968. 22024511 10.3851/IMP1842

[28] Chen DS, Kuo GC, Sung JL, Lai MY, Sheu JC, et al (1990) Hepatitis C virus infection in an area hyperendemic for hepatitis B and chronic liver disease: the Taiwan experience. J Infect Dis 162: 817–822. 2169497 10.1093/infdis/162.4.817

[29] Chen CH, Yang PM, Huang GT, Lee HS, Sung JL, et al (2007) Estimation of seroprevalence of hepatitis B virus and hepatitis C virus in Taiwan from a large-scale survey of free hepatitis screening participants. J Formos Med Assoc 106: 148–155. 17339159 10.1016/S0929-6646(09)60231-X

[30] Bureau of National Health Insurance, Department of Health, Executive Yuan, Taiwan. (2012) Universal Health Coverage in Taiwan. Available: http://www.nhi.gov.tw/Resource/webdata/21717_1_20120808UniversalHealthCoverage.pdf. Accessed 2013 May 20.

[31] Omata M, Lesmana LA, Tateishi R, Chen PJ, Lin SM, et al (2010) Asian Pacific Association for the Study of the Liver consensus recommendations on hepatocellular carcinoma. Hepatology international 4: 439–474. 20827404 10.1007/s12072-010-9165-7 PMC2900561

[32] Altman DG (1998) Confidence intervals for the number needed to treat. Bmj 317: 1309–1312. 9804726 10.1136/bmj.317.7168.1309 PMC1114210

[33] Morgan TR, Ghany MG, Kim HY, Snow KK, Shiffman ML, et al (2010) Outcome of sustained virological responders with histologically advanced chronic hepatitis C. Hepatology. 52: 833–844. 10.1002/hep.23744 PMC2932862 20564351

[34] Yu ML, Huang CF, Dai CY, Huang JF, Chuang WL (2007) Long-term effects of interferon-based therapy for chronic hepatitis C. Oncology. 72 Suppl 116–23. 10.1159/000111703 18087178

[35] Hung CH, Lee CM, Lu SN, Wang JH, Hu TH, et al (2006) Long-term effect of interferon alpha-2b plus ribavirin therapy on incidence of hepatocellular carcinoma in patients with hepatitis C virus-related cirrhosis. Journal of Viral Hepatitis 13: 409–414. 16842444 10.1111/j.1365-2893.2005.00707.x

[36] Younossi Z, Kallman J, Kincaid J (2007) The effects of HCV infection and management on health-related quality of life. Hepatology 45: 806–816. 17326207 10.1002/hep.21565

[37] Bonis PA, Ioannidis JP, Cappelleri JC, Kaplan MM, Lau J (1997) Correlation of biochemical response to interferon alfa with histological improvement in hepatitis C: a meta-analysis of diagnostic test characteristics. Hepatology 26: 1035–1044. 9328332 10.1002/hep.510260436

[38] Ingiliz P, Valantin MA, Preziosi P, Finzi L, Pais R, et al (2012) Influence of interferon-based therapy on liver fibrosis progression in HIV/HCV coinfected patients: a retrospective repeated liver biopsy analysis. Journal of Hepatology 56: 49–54. 21781946 10.1016/j.jhep.2011.05.028

[39] Asahina Y, Tsuchiya K, Tamaki N, Hirayama I, Tanaka T, et al (2010) Effect of aging on risk for hepatocellular carcinoma in chronic hepatitis C virus infection. Hepatology 52: 518–527. 20683951 10.1002/hep.23691

