# Supplementary material for: Interferon-Based Therapy Decreases Risks of Hepatocellular Carcinoma and Complications of Cirrhosis in Chronic Hepatitis C Patients
Source: PLoS One. 2013 Jul 23;8(7):e70458. doi: 10.1371/journal.pone.0070458 (PMC3720923; doi:10.1371/journal.pone.0070458)
Supplement: Table S1 — International Classification of Diseases, Ninth Revision, Clinical Modification (ICD-9-CM) codes used in the study. (DOC) [file pone.0070458.s001.doc]

**Table S**1. The International Classification of Diseases, Ninth Revision, Clinical Modification (ICD-9-CM) codes used in the study.

| **Diagnosis of Hepatitis C virus (HCV) infection** | **ICD-9-CM codes** |
| --- | --- |
| HCV with hepatic coma | 070.41 |
| Chronic hepatitis C (CHC) with hepatic coma | 070.44 |
| HCV without mention of hepatic coma | 070.51 |
| CHC without mention of hepatic coma | 070.54 |
| Viral HCV carrier | V02.62 |

| **Clinical outcomes** | **ICD-9-CM codes** |
| --- | --- |
| Hepatocellular carcinoma | 155 |
| Cirrhosis | 571.2, 571.5, 571.6 |
| Hepatic encephalopathy | 572.2 |
| Esophageal varices bleeding | 456.0, 456.20 |
| Ascites | 789.5 |

| **Comorbidities** | **ICD-9-CM codes** |
| --- | --- |
| Diabetes mellitus | 250 |
| Obesity | 278, 278.0, 278.00, 278.01 |
| Human immunodeficiency virus infection | 042, V08, 079.53 |
| Alcohol intoxication | 303.0, 303.9, 305.0 |
| Ischemic heart diseases | 410-414 |
| Cerebrovascular disease | 430-438 |
| Chronic obstructive pulmonary diseases | 490-496 |
| Chronic renal failure | 585, 403.01, 403.11, 403.91, 404.02, 404.03, 404.12, 404.13, 404.92, 404.93 |
| Hepatitis B virus infection | 070.2, 070.3, V02.61 |
