# Supplementary material for: Interferon-Based Therapy Decreases Risks of Hepatocellular Carcinoma and Complications of Cirrhosis in Chronic Hepatitis C Patients
Source: PLoS One. 2013 Jul 23;8(7):e70458. doi: 10.1371/journal.pone.0070458 (PMC3720923; doi:10.1371/journal.pone.0070458)
Supplement: Table S2 — Interferon-based regimens for treatment of hepatitis C viral infection in Taiwan. (DOC) [file pone.0070458.s002.doc]

**Table S2. Interferon-based regimens for treatment of hepatitis C viral infection in Taiwan**

| **Type** | **Interferon-based regimens** |
| --- | --- |
| 1 | Interferon alpha-2a |
| 2 | Interferon alpha-2a + Ribavirin |
| 3 | Interferon alpha-2b |
| 4 | Interferon alpha-2b |
| 5 | Interferon alfacon-1 + Ribavirin |
| 6 | Peginterferon alfa-2a + Ribavirin |
| 7 | Peginterferon alfa-2a |
| 8 | Peginterferon alfa-2a + Ribavirin |
| 9 | Peginterferon alpha-2b |
| 10 | Peginterferon alpha-2b + Ribavirin |

These drug combinations were prescribed serially with a period of therapy between 4 and 6 months.
