# Supplementary material for: Interferon-Based Therapy Decreases Risks of Hepatocellular Carcinoma and Complications of Cirrhosis in Chronic Hepatitis C Patients
Source: PLoS One. 2013 Jul 23;8(7):e70458. doi: 10.1371/journal.pone.0070458 (PMC3720923; doi:10.1371/journal.pone.0070458)
Supplement: Table S3 — Distribution of the loss to follow-up rate for each clinical outcome. (DOC) [file pone.0070458.s003.doc]

**Table S3. Distribution of the loss to follow-up rate for each clinical outcome.**

**a.**

|  | **IBT** | | ***P* value ^a^** |
| --- | --- | --- | --- |
|  | **Yes (n=457)** | **No (n=9,601)** |  |
| Withdraw insurance, n (%) |  |  | <0.001 |
| Yes | 5 (1.1) | 1305 (13.6) |  |
| No | 452 (98.9) | 8296 (86.4) |  |

**b.**

|  | **IBT** | | ***P* value ^a^** |
| --- | --- | --- | --- |
|  | **Yes (n=515)** | **No (n=10,253)** |  |
| Withdraw insurance, n (%) |  |  | <0.001 |
| Yes | 12 (2.3) | 1679 (16.4) |  |
| No | 503 (97.7) | 8574 (83.6) |  |

**c.**

|  | **IBT** | | ***P* value ^a^** |
| --- | --- | --- | --- |
|  | **Yes (n=518)** | **No (n=10,244)** |  |
| Withdraw insurance, n (%) |  |  | <0.001 |
| Yes | 12 (2.3) | 1653 (16.1) |  |
| No | 506 (97.7) | 8591 (83.9) |  |

**d.**

|  | **IBT** | | ***P* value ^a^** |
| --- | --- | --- | --- |
|  | **Yes (n=516)** | **No (n=10,126)** |  |
| Withdraw insurance, n (%) |  |  | <0.001 |
| Yes | 12 (2.3) | 1568 (15.5) |  |
| No | 504 (97.7) | 8558 (84.5) |  |

**e.**

|  | **IBT** | | ***P* value ^a^** |
| --- | --- | --- | --- |
|  | **Yes (n=373)** | **No (n=8,591)** |  |
| Withdraw insurance, n (%) |  |  | <0.001 |
| Yes | 3 (0.8) | 909 (10.6) |  |
| No | 370 (99.2) | 7682 (89.4) |  |

**f.**

|  | **IBT** | | ***P* value ^a^** |
| --- | --- | --- | --- |
|  | **Yes (n=509)** | **No (n=9,857)** |  |
| Withdraw insurance, n (%) |  |  | <0.001 |
| Yes | 12 (2.4) | 1395 (14.2) |  |
| No | 497 (97.6) | 8462 (85.8) |  |

^a^ Tested by the Chi-square test. IBT, interferoSn-based therapy.
