# Supplementary material for: Interferon-Based Therapy Decreases Risks of Hepatocellular Carcinoma and Complications of Cirrhosis in Chronic Hepatitis C Patients
Source: PLoS One. 2013 Jul 23;8(7):e70458. doi: 10.1371/journal.pone.0070458 (PMC3720923; doi:10.1371/journal.pone.0070458)
Supplement: Table S4 — Total person-years of follow-up for each of the clinical outcomes stratified by treatment group. (DOC) [file pone.0070458.s004.doc]

**Table S4**. Total person-years of follow-up for each of the clinical outcomes stratified by treatment group.

|  | Follow-up (person-years) | | | |
| --- | --- | --- | --- | --- |
|  | Treatment Group | | | |
| Clinical Outcome | IBT | | No | |
| Hepatocellular Carcinoma | 2244.7 | | 45972.8 | |
| Esophageal varices bleeding | 2547.0 | | 50124.4 | |
| Hepatic encephalopathy | 2556.7 | | 49737.4 | |
| Ascites | 2556.0 | | 49080.0 | |
| Cirrhosis | 1756.0 | | 39968.8 | |
| Any cirrhosis complication | 2501.4 | | 47253.0 | |
|  | IBT ≧ 6m | IBT < 6m | | No |
| Hepatocellular Carcinoma | 1223.1 | 1021.6 | | 45972.8 |
| Esophageal varices bleeding | 1412.0 | 1135.0 | | 50124.4 |
| Hepatic encephalopathy | 1416.3 | 1140.4 | | 49737.4 |
| Ascites | 1423.1 | 1132.8 | | 49080.0 |
| Cirrhosis | 944.0 | 811·9 | | 39968.8 |
| Any cirrhosis complication | 1389.5 | 1111.9 | | 47253.0 |

IBT, interferon-based therapy.
