# Supplementary material for: Interferon-Based Therapy Decreases Risks of Hepatocellular Carcinoma and Complications of Cirrhosis in Chronic Hepatitis C Patients
Source: PLoS One. 2013 Jul 23;8(7):e70458. doi: 10.1371/journal.pone.0070458 (PMC3720923; doi:10.1371/journal.pone.0070458)
Supplement: Table S5 — Median years of follow-up for each of the clinical outcomes according to treatment group. (DOC) [file pone.0070458.s005.doc]

**Table S5**. Median years of follow-up for each of the clinical outcomes according to treatment group.

|  | Median follow-up (Range) (years) | | | |
| --- | --- | --- | --- | --- |
|  | Treatment Group | | | |
| Clinical Outcome | IBT | | No | |
| Hepatocellular Carcinoma | 4.9 (1.0 – 9.0) | | 4.6 (0.5 – 9.2) | |
| Esophageal varices bleeding | 4.8 (1.0 – 9.0) | | 4.8 (0.5 – 9.2) | |
| Hepatic encephalopathy | 4.8 (1.0 – 9.0) | | 4.7 (0.5 – 9.2) | |
| Ascites | 4.9 (1.0 – 9.0) | | 4.7 (0.5 – 9.2) | |
| Cirrhosis | 4.6 (0.5 – 9.0) | | 4.5 (0.5 – 9.2) | |
| Any cirrhosis complication | 4.8 (1.0 – 9.0) | | 4.6 (0.5 – 9.2) | |
|  | IBT ≧ 6m | IBT ≧ 3m | | No |
| Hepatocellular Carcinoma | 4.8 (1.2 – 9.0) | 4.9 (1.0 – 9.0) | | 4.6 (0.5 – 9.2) |
| Esophageal varices bleeding | 4.8 (1.2 – 9.0) | 5.1 (1.0 – 9.0) | | 4.8 (0.5 – 9.2) |
| Hepatic encephalopathy | 4.7 (1.2 – 9.0) | 5.0 (1.0 – 9.0) | | 4.7 (0.5 – 9.2) |
| Ascites | 4.8 (1.2 – 9.0) | 5.1 (1.0 – 9.0) | | 4.7 (0.5 – 9.2) |
| Cirrhosis | 4.5 (0.8 – 9.0) | 4.7 (0.5 – 9.0) | | 4.5 (0.5 – 9.2) |
| Any cirrhosis complication | 4.7 (1.2 – 9.0) | 5.0 (1.0 – 9.0) | | 4.6 (0.5 – 9.2) |

IBT, interferon-based therapy.
