# Supplementary material for: Interferon-Based Therapy Decreases Risks of Hepatocellular Carcinoma and Complications of Cirrhosis in Chronic Hepatitis C Patients
Source: PLoS One. 2013 Jul 23;8(7):e70458. doi: 10.1371/journal.pone.0070458 (PMC3720923; doi:10.1371/journal.pone.0070458)
Supplement: Table S6 — The number of ultrasonography and a-fetoprotein examinations between IBT treated and non-treated cohorts for each clinical outcome. (DOC) [file pone.0070458.s006.doc]

**Table S6. The number of ultrasonography and a-fetoprotein examinations between IBT treated and non-treated cohorts for each clinical outcome.**

**a.**

|  | **IBT** | | ***P* value ^a^** |
| --- | --- | --- | --- |
|  | **Yes (n=457)** | **No (n=9,601)** |  |
| Ultrasonography, n (%) |  |  | <0.001 |
| Yes | 454 (99.3) | 8335 (86.8) |  |
| No | 3 (0.7) | 1266 (13.2) |  |
| a-fetoprotein, n (%) |  |  | <0.001 |
| Yes | 446 (97.6) | 6779 (70.6) |  |
| No | 11 (2.4) | 2822 (29.4) |  |
| Ultrasonography, (mean ± SD) | 9.6 ± 6.0 | 4.9 ± 5.8 | <0.001 |
| alpha-fetoprotein, (mean ± SD) | 11.0 ± 8.9 | 4.1 ± 6.3 | <0.001 |

**b.**

|  | **IBT** | | ***P* value ^a^** |
| --- | --- | --- | --- |
|  | **Yes (n=515)** | **No (n=10,253)** |  |
| Ultrasonography, n (%) |  |  | <0.001 |
| Yes | 512 (99.4) | 8933 (87.1) |  |
| No | 3 (0.6) | 1320 (12.9) |  |
| a-fetoprotein, n (%) |  |  | <0.001 |
| Yes | 504 (97.9) | 7367 (71.9) |  |
| No | 11 (2.1) | 2886 (28.1) |  |
| Ultrasonography, (mean ± SD) | 10.0 ± 6.4 | 5.0 ± 5.8 | <0.001 |
| a-fetoprotein, (mean ± SD) | 11.8 ± 9.3 | 4.4 ± 6.8 | <0.001 |

**c**.

|  | **IBT** | | ***P* value ^a^** |
| --- | --- | --- | --- |
|  | **Yes (n=518)** | **No (n=10,244)** |  |
| Ultrasonography, n (%) |  |  | <0.001 |
| Yes | 515 (99.4) | 8938 (87.3) |  |
| No | 3 (0.6) | 1306 (12.7) |  |
| a-fetoprotein, n (%) |  |  | <0.001 |
| Yes | 507 (97.9) | 7372 (72) |  |
| No | 11 (2.1) | 2872 (28) |  |
| Ultrasonography, (mean ± SD) | 10.0 ± 6.4 | 5.0 ± 5.8 | <0.001 |
| a-fetoprotein, (mean ± SD) | 11.9 ± 9.6 | 4.5 ± 6.9 | <0.001 |

**d.**

|  | **IBT** | | ***P* value ^a^** |
| --- | --- | --- | --- |
|  | **Yes (n=516)** | **No (n=10,126)** |  |
| Ultrasonography,, n (%) |  |  | <0.001 |
| Yes | 513 (99.4) | 8824 (87.1) |  |
| No | 3 (0.6) | 1302 (12.9) |  |
| a-fetoprotein , n (%) |  |  | <0.001 |
| Yes | 505 (97.9) | 7269 (71.8) |  |
| No | 11 (2.1) | 2857 (28.2) |  |
| Ultrasonography, (mean ± SD) | 10.0 ± 6.4 | 5.0 ± 5.8 | <0.001 |
| a-fetoprotein, (mean ± SD) | 12.0 ± 9.7 | 4.5 ± 6.9 | <0.001 |

**e.**

|  | **IBT** | | ***P* value ^a^** |
| --- | --- | --- | --- |
|  | **Yes (n=373)** | **No (n=8,591)** |  |
| Ultrasonography, n (%) |  |  | <0.001 |
| Yes | 370 (99.2) | 7373 (85.8) |  |
| No | 3 (0.8) | 1218 (14.2) |  |
| a-fetoprotein, n (%) |  |  | <0.001 |
| Yes | 362 (97.1) | 5938 (69.1) |  |
| No | 11 (2.9) | 2653 (30.9) |  |
| Ultrasonography, (mean ± SD) | 8.9 ± 5.9 | 4.6 ± 5.4 | <0.001 |
| a-fetoprotein, (mean ± SD) | 10.1 ± 8.3 | 3.9 ± 6.2 | <0.001 |

**f.**

|  | **IBT** | | ***P* value ^a^** |
| --- | --- | --- | --- |
|  | **Yes (n=509)** | **No (n=9,857)** |  |
| Ultrasonography, n (%) |  |  | <0.001 |
| Yes | 506 (99.4) | 8576 (87) |  |
| No | 3 (0.6) | 1281 (13) |  |
| a-fetoprotein, n (%) |  |  | <0.001 |
| Yes | 498 (97.8) | 7052 (71.5) |  |
| No | 11 (2.2) | 2805 (28.5) |  |
| Ultrasonography, (mean ± SD) | 9.9 ± 6.4 | 5.0 ± 5.8 | <0.001 |
| a-fetoprotein, (mean ± SD) | 11.8 ± 9.4 | 4.4 ± 6.9 | <0.001 |

^a^ Tested by the Mann-Whitney U test and the Chi-square test. IBT, interferon-based therapy.
