# Supplementary material for: Interferon-Based Therapy Decreases Risks of Hepatocellular Carcinoma and Complications of Cirrhosis in Chronic Hepatitis C Patients
Source: PLoS One. 2013 Jul 23;8(7):e70458. doi: 10.1371/journal.pone.0070458 (PMC3720923; doi:10.1371/journal.pone.0070458)
Supplement: Table S7 — Incidence of clinical outcomes according to treatment group. (DOC) [file pone.0070458.s007.doc]

**Table S7**. Incidence of clinical outcomes according to treatment group.

|  | Treatment Group | | | |
| --- | --- | --- | --- | --- |
| Clinical Outcome, n (%) | IBT | | No | |
| Hepatocellular Carcinoma | 18 (3.9) | | 542 (5.6) | |
| Esophageal varices bleeding | 8 (1.6) | | 224 (2.2) | |
| Hepatic encephalopathy | 11 (2.1) | | 365 (3.6) | |
| Ascites | 8 (1.6) | | 372 (3.7) | |
| Cirrhosis | 30 (8.0) | | 783 (9.1) | |
| Any cirrhosis complication | 15 (2.9) | | 529 (5.4) | |
| Clinical Outcome, n (%) | IBT ≧ 6m | IBT < 6m | | No |
| Hepatocellular Carcinoma | 8 (3.1) | 10 (4.9) | | 542 (5.6) |
| Esophageal varices bleeding | 6 (2.1) | 2 (0.9) | | 224 (2.2) |
| Hepatic encephalopathy | 4 (1.4) | 7 (3.1) | | 365 (3.6) |
| Ascites | 5 (1.7) | 3 (1.3) | | 372 (3.7) |
| Cirrhosis | 16 (7.8) | 14 (8.3) | | 783 (9.1) |
| Any cirrhosis complication | 9 (3.1) | 6 (2.7) | | 529 (5.4) |

IBT, interferon-based therapy.
