# Supplementary material for: Interferon-Based Therapy Decreases Risks of Hepatocellular Carcinoma and Complications of Cirrhosis in Chronic Hepatitis C Patients
Source: PLoS One. 2013 Jul 23;8(7):e70458. doi: 10.1371/journal.pone.0070458 (PMC3720923; doi:10.1371/journal.pone.0070458)
Supplement: Table S8 — The number needed to treat for each clinical outcome. (DOC) [file pone.0070458.s008.doc]

**Table S8. The number needed to treat for each clinical outcome.**

| **Outcome** | **NNTB** | **95% CI** |
| --- | --- | --- |
| Hepatocellular Carcinoma | 58.6 | NNTH 224.7 to ∞ to NNTB 25.9 |
| Esophageal varices bleeding | 158.4 | NNTH 153.0 to ∞ to NNTB 52.2 |
| Hepatic encephalopathy | 69.5 | NNTH 552.3 to ∞ to NNTB 32.7 |
| Ascites | 47.1 | NNTB 26.6 to ∞ to NNTB 207.3 |
| Cirrhosis | 93.3 | NNTH 52.5 to ∞ to NNTB 24.7 |
| Any cirrhosis complication | 41.3 | NNTB 22.7 to ∞ to NNTB 230.6 |

NNTB, number needed to benefit; NNTH, number needed to harm.
